# *IL1B-CGTC* haplotype is associated with colorectal cancer in admixed individuals with increased African ancestry

**DOI:** 10.1038/srep41920

**Published:** 2017-02-03

**Authors:** María Carolina Sanabria-Salas, Gustavo Hernández-Suárez, Adriana Umaña-Pérez, Konrad Rawlik, Albert Tenesa, Martha Lucía Serrano-López, Myriam Sánchez de Gómez, Martha Patricia Rojas, Luis Eduardo Bravo, Rosario Albis, José Luis Plata, Heather Green, Theodor Borgovan, Li Li, Sumana Majumdar, Jone Garai, Edward Lee, Hassan Ashktorab, Hassan Brim, Li Li, David Margolin, Laura Fejerman, Jovanny Zabaleta

**Affiliations:** 1 Subdirección de Investigaciones, Instituto Nacional de Cancerología de Colombia, Bogotá D.C., Colombia; 2 Departamento de Química, Universidad Nacional de Colombia, Bogotá D.C., Colombia; 3 The Roslin Institute and Royal (Dick) School of Veterinary Studies, University of Edinburgh, UK; 4 MRC-Human Genetics Unit, University of Edinburgh, UK; 5 Escuela de Salud Pública, Universidad del Valle, Cali, Colombia; 6 Servicio de Gastroenterología, Instituto Nacional de Cancerología de Colombia, Bogotá D.C., Colombia; 7 Fundación Oftalmológica de Santander, Bucaramanga, Colombia; 8 Ochsner Clinic Foundation, New Orleans, LA, US; 9 Stanley S. Scott Cancer Center, Louisiana State University Health Sciences Center, New Orleans, LA, US; 10 Department of Pathology & Cancer Center, Howard University College of Medicine, Washington, D.C., US; 11 Department of Medicine, Division of General Internal Medicine, Institute for Human Genetics and Helen Diller Family Comprehensive Cancer Center, University of California, San Francisco, CA, US; 12 Department of Pediatrics, Louisiana State University Health Sciences Center, New Orleans, LA, US

## Abstract

Single-nucleotide polymorphisms (SNPs) in cytokine genes can affect gene expression and thereby modulate inflammation and carcinogenesis. However, the data on the association between SNPs in the interleukin 1 beta gene (*IL1B*) and colorectal cancer (CRC) are conflicting. We found an association between a 4-SNP haplotype block of the *IL1B* (-3737C/-1464G/-511T/-31C) and CRC risk, and this association was exclusively observed in individuals with a higher proportion of African ancestry, such as individuals from the Coastal Colombian region (odds ratio, OR 2.06; 95% CI 1.31–3.25; *p* < 0.01). Moreover, a significant interaction between this CRC risk haplotype and local African ancestry dosage was identified in locus *2q14 (p* = 0.03). We conclude that Colombian individuals with high African ancestry proportions at locus *2q14* harbour more *IL1B-CGTC* copies and are consequently at an increased risk of CRC. This haplotype has been previously found to increase the *IL1B* promoter activity and is the most frequent haplotype in African Americans. Despite of limitations in the number of samples and the lack of functional analysis to examine the effect of these haplotypes on CRC cell lines, our results suggest that inflammation and ethnicity play a major role in the modulation of CRC risk.

Colorectal cancer (CRC) is considered a major public health problem and is the fourth most common cancer and the fifth leading cause of cancer-related death in both sexes worldwide^1^. Thus, studies of the effects of non-genetic^2^,^3^ and genetic risk factors on CRC development^4^ have important implications for understanding the aetiology of this disease.

Inflammation is a hallmark of cancer^5^ and has been particularly associated with genetic instability and stromal mechanisms that affect the CRC tumour microenvironment, including angiogenesis, invasion and metastasis^6^. Specifically, interleukin 1 beta protein (IL-1 beta) plays a critical role as a pro-inflammatory cytokine in colon inflammation and carcinogenesis^7^ by up-regulating cyclooxygenase-2 (COX-2) and prostaglandin E_2_ (PGE2) via several signalling pathways^8^,^9^; furthermore, SNPs within the promoter region of the gene can modulate the IL-1 beta levels and this modulation depends upon the haplotype context^10^. However, the frequencies of these haplotypes vary by population^10^, which may partly explain the disparities observed in CRC incidence between African Americans and U.S. Non-Hispanic Whites^11^,^12^.

A heterogeneous risk pattern for CRC mortality across Colombian populations has been reported^13^, and this pattern could be explained by the influence of environmental and genetic factors^13^, including genetic ancestry. The latter is supported by the fact that Colombian populations are characterized by different three-way admixture contributions from ancestral source populations; for instance, the proportions of African ancestry are higher across cities near the Coastal compared to those located within the Andean region^14^,^15^. Moreover, African ancestry was previously shown to be associated with increased risk of CRC in individuals from Colombia^16^.

Previous genetic studies of admixed individuals have identified DNA markers linked to genetic ancestry that are associated with the risk of various types of cancer, and these markers partly explain the disparities in health between some populations groups^17^,^18^,^19^,^20^,^21^. Because recombination events can change allele and haplotype frequencies among populations, especially in cases of admixture between different ethnic groups^22^,^23^, and because the data on the effects of *IL1B* variants on cancer risk differ by study population^24^,^25^,^26^,^27^,^28^,^29^, we took advantage of the characteristic variation in the genetic structure of our admixed samples to identify the associations between adenomatous polyps (AP) as well as CRC risk with *IL1B* haplotypes and their interactions with global and local ancestry proportions in Colombians; we also analysed the variations in these effects by region. To the best of our knowledge, this study is the first to explore the association of *IL1B* haplotypes with AP and CRC risk in a genetically admixed population.

## Results

### Participants’ characteristics

Descriptive characteristics for three groups of participants, AP, CRC and controls, from the Andean and Coastal Colombian regions are summarized in Supplementary Table S1. The AP and CRC groups contained more subjects within the age range of 50 to 69 years compared to controls (68.6%, 63.8% and 45.8%, respectively; *p* < 0.01). In addition, compared to controls, the proportion of male subjects was higher in the CRC group (42.4% and 50.7%, respectively; *p* = 0.03), and a larger percentage of the AP group had a college degree or a higher education level (14.6% and 24.1%, respectively; *p* = 0.03), whereas more patients with CRC had no education or only completed primary school (34.7% and 51%, respectively; *p* < 0.01). The family history of CRC in immediate relatives, NSAID consumption or region of origin did not differ between groups.

### Differences in the allele, genotype and haplotype frequencies of *IL1B* SNPs by case-control status

We selected five *IL1B* SNPs for which conflicting results regarding their association with inflammation and neoplastic processes had been published in previous studies^24^,^25^,^26^,^27^,^28^,^29^; four of these SNPs are located within the promoter region (−3737 C > T, −1464 G > C, −511C > T and −31 T > C), and the fifth is located in the coding region (+3954 C > T). In single SNP analyses we found that *IL1B-1464CC* individuals (versus CG + GG) were protected from AP but not CRC (*p* = 0.04). No other associations were observed (Supplementary Table S2).

Given the high linkage disequilibrium (LD) between the SNPs included in the analysis and due to previous work investigating haplotypes of *IL1B* in other populations^10^, we obtained haplotypes for this region based on the 5 genotyped SNPs. The four SNPs located in the promoter region of the *IL1B* gene are in strong LD and form one haplotype block (Supplementary Fig. S1). Within this haplotype block, only five of 16 possible *IL1B* promoter haplotypes were found with a frequency greater than 0.01 (Table 1). Because the *IL1B-CCTC* haplotype (N°4) was the most frequent haplotype in our admixed sample (41%) (Table 1), it was used as the reference in all analyses. According to the unadjusted general lineal model (GLM) analysis, the haplotype *IL1B-CGTC* (N°3) was associated with an increased risk of CRC (odds ratio, OR 1.39; 95% CI 1.02–1.90; *p* = 0.03) (Table 1). In the conditional haplotype tests controlling for the *IL1B-511*/*IL1B-31* SNPs, we confirmed that the *IL1B-1464G* allele (haplotype N°3) was a risk factor for CRC compared to having the *IL1B-1464C* allele (haplotype N°4) (subnull *p* = 0.04; likelihood ratio test: chi-square = 5.63, Degrees of freedom (*df* ) = 2, *p* = 0.06). This result was concordant with the independent effect of *IL1B-1464* test (likelihood ratio test: chi-square = 4.38, *df* = 1, *p* = 0.04) (Table 1).

### Genetic structure and global ancestry estimations

**Figure 1:**
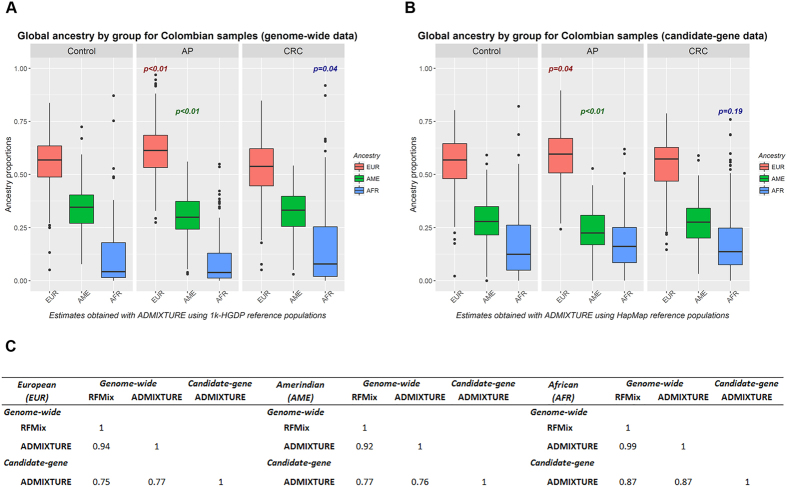
Differences in global ancestry proportions between cases and controls for genome-wide and candidate-gene genotyped samples (*P* values for the Wilcoxon rank sum Test are displayed). (**A**) Ancestry estimations per group for the genome-wide set. The difference in ancestries between the AP and control groups was significant for the European and Amerindian components (*p* < 0.01) and between the CRC and control groups for the African proportion (*p* = 0.04). (**B**) Ancestry estimations per group for the candidate-gene set. The difference in ancestries between the AP and control groups was significant for the European and Amerindian components (*p* = 0.04 and <0.01, respectively), whereas the African proportion did not significantly differ between the CRC and control groups within these candidate-gene sample sets. (**C**) Pearson’s correlation per ancestry component. The correlations between genome-wide genotyped samples obtained with RFMix and ADMIXTURE are for 393 Colombians. The correlations between genome-wide and candidate-gene sets obtained with ADMIXTURE are for 85 overlapping samples. 1k-HGDP, 1000 genomes plus Human Genome Diversity Project databases; EUR - AME - AFR corresponds to global European, Amerindian and African components; AP, adenomatous polyps; CRC, colorectal cancer.

We were able to perform Multidimensional Scaling analyses (MDS) and global ancestry estimations in Colombian samples genotyped with genome-wide and candidate-gene platforms (Supplementary Methods). In both sets, cases and controls from Colombia overlapped, and most of them scattered between the European and Amerindian/Asian reference populations but were more closely related to Europeans; in addition, a small subset of them were close to the Africans (Supplementary Fig. S2). Global ancestry proportions per individual calculated with ADMIXTURE^30^ are shown in Supplementary Fig. S2. Regarding these estimates, European, Amerindian and African ancestry proportions for genome-wide and candidate-gene sets were strongly correlated for 85 samples genotyped using both platforms, despite the use of different reference populations and numbers of SNPs in each case (Pearson’s correlation coefficients of 0.77, 0.76 and 0.87 for each component, respectively) (Fig. 1C). We also identified a strong correlation between ancestry proportions estimated using two different statistical approaches, i.e., RFMix^31^ and ADMIXTURE^30^, in the 393 samples with genome-wide genotypes (0.94, 0.92 and 0.99 for European, Amerindian and African components, respectively) (Fig. 1C).

Due to the right-skewed distribution of the African ancestry proportions in our admixed samples, we selected a non-parametric test to assess differences in global ancestry estimates among Colombian cases and controls (Fig. 1). We found significant differences in the European and Amerindian ancestry proportions between the AP and control groups for both sets of individuals (genome-wide set: *p* values < 0.01 for both components; candidate-gene set: *p* values of 0.04 and <0.01, respectively) (Fig. 1A,B). Moreover, the African ancestry proportions significantly differed between the CRC group and the control group for genome-wide samples only (*p* = 0.04) (Fig. 1A).

### *IL1B* haplotype association with AP and CRC risk adjusted for global ancestry

Among the 791 samples with both *IL1B* haplotype information and global ancestry estimations, we tested different multinomial logistic regression analyses that model phenotypes by global ancestry proportions (Supplementary Table S3). The best and least complex model according to the Akaike Information Criterion (AIC) was model 13, which included European and African ancestries along with array, sex, age, educational level and NSAID consumption (Supplementary Table S3.1). According to this model, European ancestry was only associated with AP risk (OR 1.98; 95% CI 1.35–2.91; *p* < 0.01), whereas African ancestry was associated with both AP (OR 1.12; 95% CI 1.03–1.22; *p* = 0.01) and CRC risk (OR 1.10; 95% CI 1.03–1.18; *p* = 0.01). Some of the AP and CRC risk was also explained by age, educational level and NSAID consumption (Supplementary Table S3).

Results from the unadjusted and adjusted GLM analyses to evaluate the effect of *IL1B* haplotypes on AP and CRC risk among these 791 Colombian samples are shown in Table 2. We found that haplotype *IL1B-TGCT* (N°5) was associated with an increased risk of AP in the unadjusted (OR 1.45; 95% CI 1.05–2.00; *p* = 0.02) and adjusted model (OR 1.40; 95% CI 0.98–2.00; *p* = 0.06) (Table 2). Furthermore, the association of the *IL1B-CGTC* (N°3) haplotype and CRC risk remained in both the unadjusted (OR 1.55; 95% CI 1.09–2.20; *p* = 0.02) and the adjusted model (OR 1.46; 95% CI 0.99–2.14; *p* = 0.06) (Table 2).

To identify differences in the effect of these haplotypes on the AP and CRC risk for each Colombian region, we used the same stratification method as that described in Supplementary Fig. S3. The latter showed that people from the Coast have higher African ancestry than those from the Andean region of the country. In this analysis, we saw a trend in the association of the *IL1B-TGCT* (N°5) haplotype with AP in both regions, Andean (95% CI 0.96–2.40) and Coastal (95% CI 0.93–2.49) (Table 3). Interestingly, we found that the association between the *IL1B-CGTC* (N°3) haplotype and CRC risk was exclusive to Colombians from the Coast (OR 2.06; 95% CI 1.31–3.25; *p* < 0.01) (Table 3).

### *IL1B* haplotype frequencies among self-reported African American CRC cases and reference populations

The *IL1B-CGTC* (N°3) haplotype found to be associated with an increased risk of CRC in Colombian subjects from the Coastal region was also the most frequent haplotype in the self-reported African American CRC cases from this study and the African Americans from the Atherosclerosis Risk Communities Cohort (ARIC)^10^ (Supplementary Table S4). In addition, this haplotype was the least frequent haplotype in Non-Hispanic White populations from the US^10^. We used information available on HapMap3^32^ via Haploview^33^, including a simplified haplotype configuration (with *IL1B-511* and *IL1B-31* SNPs) and found similarities in the haplotype frequencies between the Colombian control group, HapMap3 African Americans^32^ and African Americans from the ARIC study^10^ (Supplementary Table S5).

### Role of locus-specific ancestry and *IL1B* risk haplotypes on CRC and AP

**Figure 2:**
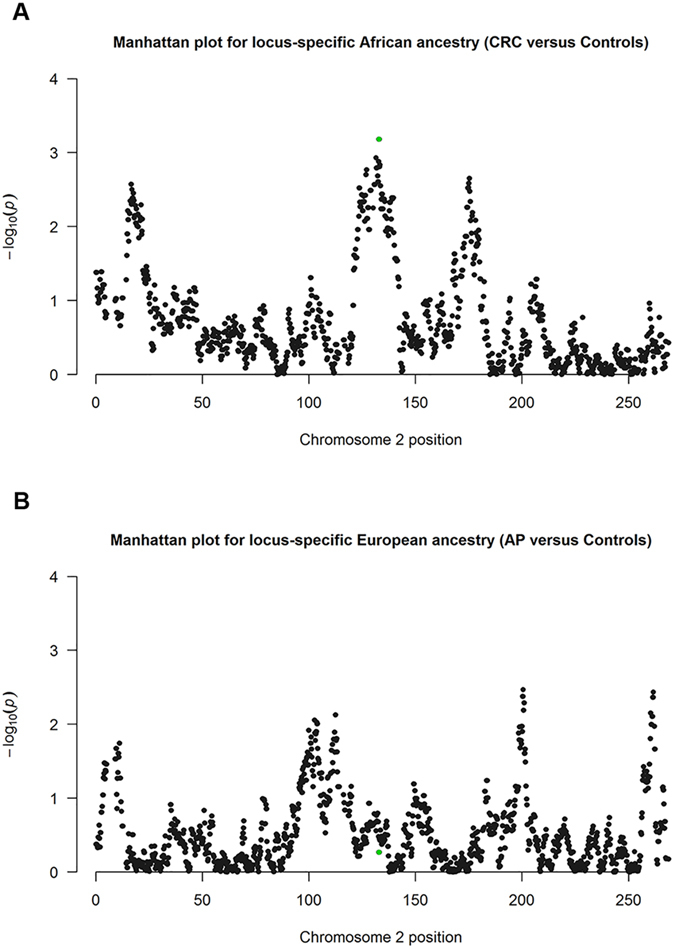
Manhattan plots of the −log10 (*P*-values) from adjusted GLM analyses that model phenotypes by local ancestry dosage per marker along chromosome 2. (**A**) Plot of the differences in local African ancestry copies between the CRC and control groups. Green overlapping dots are SNPs within the *2q14* region, which holds the *IL1B* gene (FDR corrected *p = *0.09). (**B**) Plot of the differences in local European ancestry copies between the AP and control groups. Green overlapping dots are the SNPs in the *2q14* region. CRC, colorectal cancer; AP, adenomatous polyps; FDR, false discovery rate.

The association of *IL1B* haplotypes and locus-specific ancestry with the risk of AP and CRC were analysed using only the 393 Colombian samples for which the aforementioned information was available; these samples represented only 50% of the 791 sample used previously in the adjusted regression analyses. We saw great variations in the level of excess of local African and European ancestries per marker in chromosome 2 for the CRC and AP groups relative to the control group (Supplementary Fig. S4), and the corresponding −log_10_ (*P*-values) obtained for these differences in the GLM analyses adjusted by sex, age, educational level and global ancestries are shown in Fig. 2.

Interestingly, the 18 SNPs located in the selected *2q14* region exhibited the largest differences in African ancestry dosage between the CRC group and the control group (overlapped green dots, *p = *6.58 × 10^−4^; false discovery rate, FDR, corrected *p = *0.09) (Fig. 2A); although these differences are significant at a nominal level, after correction for multiple testing the association is not significant at the 5% level but it is suggestive (*p* < 0.1).

**Figure 3:**
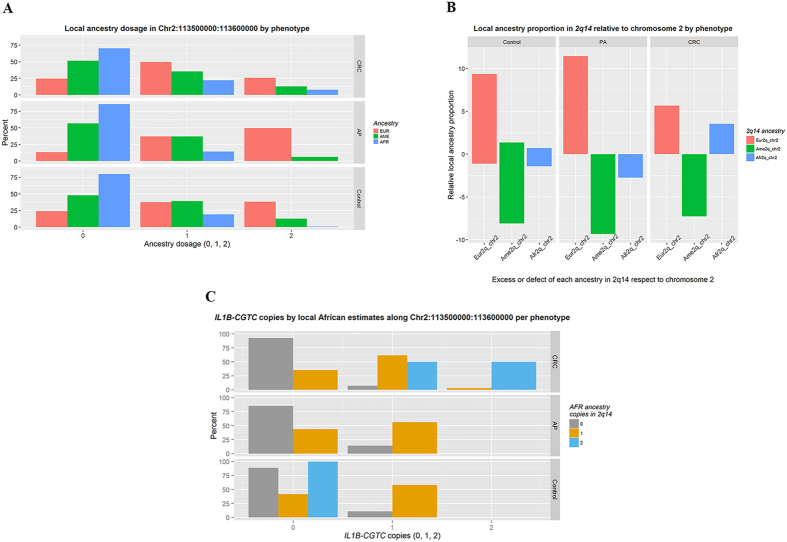
Ancestry dosage and *IL1B-CGTC* haplotype copies in Colombian samples by phenotype. (**A**) Ancestry dosage in Colombian samples by phenotype. This analysis included 393 samples with available locus ancestry estimates. An ancestry dosage of 0, 1 or 2 corresponds to the number of specific ancestry copies in the selected 100000-bp region at locus 2*q14* (Chr2:113500000:113600000) that contains the *IL1B* gene. (**B**) Local ancestry proportions in locus *2q14* relative to the average locus-specific ancestry across all loci in chromosome 2 by phenotype. (**C**) Percentage of *IL1B-CGTC* haplotype copies (0, 1 or 2) within each local African ancestry dosage group (0, 1 or 2). CRC, colorectal cancer; AP, adenomatous polyps; Chr2, chromosome 2; EUR - AME - AFR, corresponds to European, Amerindian and African ancestries; Eur2q_chr2 - Ame2q_chr2 - Afr2q_chr2, corresponds to European, Amerindian or African locus specific ancestry minus their respective ancestry proportion for all of chromosome 2.

To better understand this result, we plotted each ancestry dosage within the *2q14* region by phenotype and found that the CRC group contains a higher proportion of African ancestry dosage (1 or 2 copies) than the AP and control groups (Fig. 3A). Moreover, 50% of the CRC samples with two copies of African ancestry carried two copies of the *IL1B-CGTC* haplotype, and the remaining 50% carried at least one copy (Fig. 3C).

Table 4 shows the multinomial logistic regressions conducted to evaluate the effect of global and locus-specific African ancestry on the risk of CRC, including the *IL1B-CGTC* haplotype copies, adjusted by sex, age, educational level, NSAID consumption and family history of CRC. We found that both global and locus-specific African ancestries were individually associated with an increased risk of CRC (Model 1a and 2a, respectively). When we included the main effects of both variables in the same model, the association with CRC remained significant only for locus-specific African ancestry (OR 3.40; 95% CI 1.05–10.98; *p* = 0.04; Model 3a). Although we found that the *IL1B-CGTC* haplotype was associated with CRC risk in Colombians, especially individuals from the Coastal region characterized by having a higher African ancestry, our results for this small set of 393 individuals only suggests this association (95% CI 0.88–2.82; Model 4a). Remarkably, the interaction of *IL1B-CGTC* haplotype copies with locus-specific African ancestry was associated with CRC (*p* = 0.03; Model 6a); nevertheless, when testing only the main effects of all three variables, only locus-specific African ancestry was found to be associated with CRC (OR 3.58; 95% CI 0.97–13.30; *p* = 0.06; Model 7a).

For European ancestry dosage, none of the comparisons revealed significant differences or suggestive *p* values in the adjusted GLM analysis between the AP and control groups after correction for multiple testing, including locus *2q14* (overlapped green dots; Fig. 2B). These results are consistent with those displayed in Fig. 3B, which shows the excess or defect of each locus-specific ancestry within locus *2q14* with respect to the average of each ancestry along chromosome 2.

We also used adjusted multinomial logistic regressions to evaluate AP risk, including global and locus-specific European ancestry, and copies of the *IL1B-TGCT* haplotype (Table 4). We found that AP risk was mainly explained by the effect of global European ancestry (OR 1.91; 95% CI 1.17–3.13; *p* = 0.01; Model 7b) and that the data suggested an association with AP risk for the *IL1B-TGCT* haplotype copies (95% CI 0.83–2.66; Model 4b). As already observed in the multimarker regression analysis for AP (Fig. 2B), the European ancestry proportion in *2q14* does not play an important role in AP risk (Model 7b, Table 4).

## Discussion

We found that AP is less common among recessive carriers of the *IL1B-1464C* allele. Furthermore, the risk of CRC significantly differed between carriers of the *IL1B-1464G* allele and the *IL1B-1464C* allele, when considering the haplotype context (N°3 versus N°4). Our results and the literature support the potential effect of this variant in preventing neoplastic changes in some tissues because the *IL1B-1464G*/*C* SNP flanks a putative binding site for the proteins DBP, C/EBP alpha and Pit-1a, and the *C* allele shows a lower transcriptional activity in haplotype context with *IL1B-511T* and *IL1B-31C*^10^,^34^. Therefore, accounting for haplotype context is important in association studies.

Although increased IL-1 beta production has also been reported for other SNPs in the promoter region of the *IL1B* gene^10^,^34^,^35^,^36^,^37^, associations of these SNPs with cancer are variable and depend on the cancer model and genetic background of the population^24^,^25^,^26^,^27^,^28^,^29^. These inconsistencies are likely related to a small effect or the low frequency of these variants in some of the studied populations, which makes the identification of any association with the disease of interest difficult. Our three-way admixed population provides an opportunity to test differences in the effect of *IL1B* haplotypes among individuals with varying degrees of admixture. Regarding these analyses, we found that the second most frequent haplotype in Colombian controls, *IL1B-TGCT*, was associated with AP risk irrespective of the region of origin. Also, our results suggest that most of the AP risk can be attributed to global European ancestry proportions instead of local European ancestry at *2q14*, meaning that non-genetic factors associated with the European component could have an important role in the risk of AP. Further genome-wide association analysis taking into account other non-genetic risk factors could help disentangle the observed association between AP risk and global European ancestry in Colombians. We did not find an effect of the *IL1B-TGCT* haplotype on CRC risk, which could be explained in part by the small sample size or the fact that this haplotype exhibits moderate transcriptional activity *in vitro*^10^, making it more suitable as a susceptibility marker of milder lesions that do not necessarily evolve to CRC.

CRC risk was consistently associated with the *IL1B-CGTC* haplotype in Colombians, especially in individuals from the Coastal region of the country, who exhibit the highest African ancestry proportions. Despite this result, an analysis of 393 individuals with local ancestry inference (LAI) data only suggested an effect of *IL1B-CGTC* on CRC risk; lack of significance for this association is likely due to the small sample size and the overall low frequency of this haplotype in Colombians (~13%). The important role of African ancestry within locus *2q14* in CRC risk was suggested by the fact that variants within this region showed the lowest *p*-values in the multimarker regression analysis along chromosome 2. Interestingly, we found a significant interaction between the *IL1B-CGTC* haplotype and local African ancestry for CRC risk. Because only local African ancestry remained significantly associated with CRC risk when we tested the main effects of the risk haplotype as well as local and global African ancestries, we conclude that additional African-related variants that could explain the risk of CRC may be located within the selected *2q14* region, and fine-mapping methods will help to identify these variants.

The relationship of the *IL1B-CGTC* haplotype with CRC risk and African ancestry identified herein corroborates previous work showing that this haplotype exhibits the highest transcriptional activity amongst four other possible *IL1B* promoter haplotypes^10^ and that it is the most frequent haplotype in African Americans^10^. Therefore, further population case-control studies among Colombians with different African ancestry proportions, seeking differences in the expression levels of IL-1 beta and its targets (such as COX-2 and PGE2) in colorectal tissues and plasma samples will help prove their utility as susceptibility markers for the risk of CRC in the general population. The identification of such markers will allow individuals who are at an increased risk to be offered effective measures to prevent this type of cancer. For example, according to the atlas of cancer mortality in Colombia published in 2010^13^, two of the cities included in this study from the Andean region, Bogotá DC and Bucaramanga, belong to two regions with 27% and 11% higher risks of CRC mortality, respectively, than the rest of the country. Moreover, whereas among the cities included from the Caribbean Coast and surrounding areas, Cartagena, Barranquilla and Santa Marta, the risk of CRC mortality is lower than in the general population, in Cali and its surroundings in Valle del Cauca located in the Pacific Coast, the risk of CRC mortality is 22% higher than that in the rest of the country^13^. Interestingly, Cali has the highest concentration of Afro-Colombians^38^.

Overall, these results are of particular interest due to previous observations about disparities in CRC risk among different population groups, such as differences between Non-Hispanic White and African American U.S. populations, with respect to inflammatory diseases and cancer development^11^,^39^. Our findings highlight the importance of conducting genetic studies in admixed populations to reveal potential population-specific susceptibility markers of risk for complex diseases.

## Methods

### Subjects

This is a multicentre, hospital-based case-control study that was conducted in six Colombian cities from the Andean (Bogotá and Bucaramanga) and Coastal regions (Cartagena, Santa Marta and Barranquilla in the Caribbean and Cali in the Pacific). These cities are characterized by differences in CRC mortality risk and ancestry proportions. A total of 306 CRC cases, 191 AP and 500 matched controls between age 30 and 74 years were enrolled. All cases were incident and confirmed by histopathology. The control group consisted of individuals without gastrointestinal symptoms attending the outpatient services of primary care units. Neither cases nor controls had a personal history of other cancers, and neither group received chemotherapy or radiotherapy. Each participant provided written informed consent. This work was approved by the Ethics Committee of the Instituto Nacional de Cancerología, Bogotá, Colombia as a *“study with greater than minimal risk”* according to guidelines established in the document “RESOLUCIÓN 8430 DE 1993” for Ethical Aspects of Human Research, (Title II, Chapter 1) published by the República de Colombia Ministerio de Salud (https://www.invima.gov.co/images/pdf/medicamentos/resoluciones/etica_res_8430_1993.pdf).

We also included samples from 177 self-reported African American patients with CRC diagnosed at the Howard University College of Medicine in Washington, D.C. and the Ochsner Clinic Foundation in New Orleans, LA for *IL1B* SNPs genotyping to compare the *IL1B* haplotype frequencies with those of Colombian samples. Because these samples were de-identified FFPE tissues and the study was retrospective, the LSUHSC IRB following the recommendations for the use of human tissues and under strictest HIPAA protocols classified this specific study as an *“Institutional Review Board (IRB) exempt”* study.

All methods were performed in accordance with the declaration of Helsinki and local relevant guidelines and regulations, as already mentioned.

### DNA extraction

DNA was extracted from 200 μl of buffy coat using the QIAamp DNA Blood Mini Kit (QIAGEN, Valencia, CA, USA) according to the manufacturer’s protocol. The DNA was resuspended in 100 μl of Ambion Nuclease-free Water (Ambion, Foster City, CA, USA) and stored at −20 °C. DNA purity and concentration were assessed using a NanoDrop 2000 (Thermo Scientific, Wilmington, DE, USA).

### *IL1B SNPs* genotyping

All subjects were genotyped for five *IL1B* SNPs using TaqMan SNP Genotyping Assays Kits (Applied Biosystems, Foster City, CA, USA) according to the manufacturer’s protocol. Four of these SNPs are located in the promoter region of the gene: −3737 C > T (rs4848306), −1464 G > C (rs1143623), 511 C > T (rs16944) and −31 T > C (rs1143627). The fifth SNP +3954 C > T (rs1143634) is located within exon 5. After PCR amplification, the genotype was determined using the Sequence Detection System (SDS) software (Applied Biosystems, Foster City, CA, USA). DNA controls with known genotype for each SNP were run in parallel. Hardy-Weinberg equilibrium for *IL1B-*genotyped SNPs was assessed using the exact test statistics *P*_*HWE*_ in PLINK^40^,^41^. All *IL1B* SNPs were in Hardy-Weinberg equilibrium (*p* > 0.05) (data not shown).

### Global ancestry estimation

We used two different platforms from Illumina®, a candidate-gene “*Cancer SNP Panel”* array that includes 1421 SNPs, and a genome-wide “*Infinium® OmniExpressExome Array”*, which includes 958178 SNPs, according to the manufacturer’s instructions, to genotype 521 and 443 Colombian samples, respectively.

Quality control (QC) and pruning steps were performed separately due to large differences in the number of markers tested within each array. These steps were based on the protocol described by Anderson *et al*.^42^ using PLINK v1.07^40^ and the R statistics v3.2.2^43^ software, as recommended. All datasets were lifted over to the human genome build 37 coordinates as necessary. After QC steps for the candidate-gene dataset (Supplementary Methods), a total of 1237 SNPs and 483 samples remained for further analysis. For genome-wide data, a total of 720815 SNPs and 415 samples remained for further analysis after QC procedures (Supplementary Methods).

We used the unsupervised model-based mode in ADMIXTURE^30^ for individual ancestry estimations using genotypes from autosomal markers and K = 3 ancestral populations individually for candidate-gene and genome-wide datasets. For candidate-gene data, reference populations from the HapMap3 project^32^ public database were used (CEU = Utah residents of Northern and Western European ancestry; LWK = Luhya in Webuye, Kenya; and CHB = Han Chinese in Beijing, China; MEX = Mexican ancestry in Los Angeles, California) (Supplementary Methods). Because the allele frequencies are similar between Asians and Amerindians, we used the CHB reference population to discriminate the Amerindian component^16^,^44^. The final merged and pruned candidate-gene database used to infer global ancestry in 483 Colombian samples included 473 overlapping SNPs. For genome-wide data, we included the genotypes of reference populations from public databases, 1000 genomes^45^ (IBS = Iberian Population in Spain and YRI = Yoruba in Ibadan, Nigeria) plus HGDP^46^ (AME = Amerindians, which includes Pima, Maya, Karitiana, Surui and Colombian Native Americans) (Supplementary Methods). The final merged and pruned genome-wide database used to infer global ancestry in 415 Colombian samples included 9663 overlapping SNPs.

After eliminating duplicates between both arrays (n = 85 samples) and selecting samples also genotyped for *IL1B* SNPs, a total of 791 Colombian samples with available global ancestry estimates remained for further adjusted regression analyses.

### Local ancestry inference (LAI)

Among all available methods for LAI^47^,^48^,^49^,^50^,^51^, we selected a discriminative approach supported by the RFMix v1.5.4 software^31^ because it performs an iterative analysis that, beginning with unadmixed panels, also utilizes information in the chromosomes of admixed individuals to infer local ancestry. This approach allows refining our knowledge of haplotype patterns in the ancestral populations and improves accuracy via expectation maximization (EM) steps^31^.

For the LAI procedures, we used a reference panel that included the same populations from 1000 genomes^45^ and HGDP^46^ databases incorporated previously to calculate global ancestry proportions. Because RFMix^31^ requires phased haplotypes input, we first phased genotypes from our genome-wide Colombian samples and HGDP^46^ data using 1000 genomes^45^ as a reference prior to the merging steps (Supplementary Methods). Specifically, we used the *segmented haplotype estimation and imputation tool*, SHAPEIT v2.778^52^, based on the modelling of stochastic processes through a Hidden Markov Model to phase genotypes. To correct for admixture in our reference populations, we performed five EM iterations, as recommended^31^.

### Statistical analysis

A descriptive analysis based on the characteristics of the 997 Colombian samples genotyped for the *IL1B* gene SNPs was performed in R^43^ using Pearson’s Chi-Squared Test to assess for differences among groups. Full model association tests between the disease and each SNP were performed in PLINK^40^. LD among all five *IL1B* SNPs was calculated using the Haploview algorithm^33^.

Global ancestry estimates from ADMIXTURE^30^ with candidate-gene or genome-wide datasets were obtained for 791 unique Colombian samples with *IL1B* haplotype information. Furthermore, the LAI results from RFMix^31^ were used to calculate global ancestry as the average locus-specific ancestry across all loci for each individual with genome-wide data. Pearson’s correlation per ancestry component was computed between all estimates. Differences in ancestry proportions between the control and CRC or AP group were assessed with the Wilcoxon rank sum test for ancestry estimates obtained with ADMIXTURE^30^.

Because the sample size in this study is at best moderate and the observed African ancestry proportions were substantially skewed, we transformed the European and African ancestry components to a symmetric distribution using a logit transformation before conducting adjusted regression analyses. In these models, Amerindian ancestry was treated as the reference, and global ancestry proportions were always corrected by the “array” variable, referring to candidate-gene or genome-wide estimates.

Multinomial logistic regressions of phenotypes, including global ancestry estimates, sex, age, educational level, city of origin, NSAID consumption and a family history of CRC as explanatory covariates, were conducted with R^43^. The AIC was used to select the best model.

The *IL1B* haplotype frequencies among Colombian and African American CRC samples were inferred in R^43^, through EM computation of haplotype probabilities with progressive insertion of loci. Conditional haplotype tests were conducted in PLINK^40^ to evaluate differences in disease risk related to haplotype context when controlling for some SNPs or when assessing for their independent effect. The odds ratio (OR) for each *IL1B* haplotype and disease risk were obtained for Colombian samples using unadjusted and adjusted GLM in R^43^ while controlling for sex, age, educational level, global ancestry proportions and array variables. GLM analyses of *IL1B* haplotypes and disease risk were conducted in a stratified analysis by region of origin, using sex, age and educational level as covariates. In all cases, the most frequent haplotype was used as the reference, and rare haplotypes (less than 0.01) were not included in the analyses.

For the 393 Colombian samples with both *IL1B* haplotypes and LAI information, we estimated global ancestry for chromosome 2 as the average locus-specific ancestry across all loci. We also selected a 100000-bp region at locus *2q14* (Chr2:113500000:113600000; 18 SNPs; build 37) that holds the *IL1B* gene to calculate locus-specific ancestry. We plotted the variation of each ancestry proportion per marker in chromosome 2 in the CRC and AP groups relative to those in the control group used as the baseline and conducted a multimarker GLM analysis for CRC risk. Specifically, the African dosage per marker at chromosome 2 corrected for sex, age, educational level and global African ancestry was used as an explanatory variable. We conducted the same analysis comparing the AP and control groups but used the European dosage per marker at chromosome 2 and global European ancestry instead. Corresponding - log10 (*P*-values) for each analysis are displayed in Manhattan plots. The computed *P*-values were adjusted for multiple comparisons using the FDR correction for 28579 test/variants along chromosome 2.

Finally, we performed multinomial logistic regression analyses that compared the respective *IL1B* risk haplotypes copies and the effect of global and locus-specific African or European ancestry of the control group with those of the CRC and AP groups, and these comparisons were adjusted for sex, age, educational level, NSAID consumption and a family history of CRC.

## Additional Information

**How to cite this article:** Sanabria-Salas, M. C. *et al. IL1B-CGTC* haplotype is associated with colorectal cancer in admixed individuals with increased African ancestry. *Sci. Rep.* **7**, 41920; doi: 10.1038/srep41920 (2017).

**Publisher's note:** Springer Nature remains neutral with regard to jurisdictional claims in published maps and institutional affiliations.

## Supplementary Material

Supplementary Information

## Figures and Tables

**Table 1:** *IL1B* haplotypes and their association with AP and CRC risk in Colombian samples.

*IL1B* Haplotypes					Total (n = 997) Frequency	Controls (n = 500) Frequency	Adenomatous Polyps (AP) (n = 191)				Colorectal Cancer (CRC) (n = 306)			
N°	−3737	−1464	−511	−31			Frequency	OR	[95% CI]	p value	Frequency	OR	[95% CI]	p value
1	C	G	C	T	0.124	0.130	0.134	1.14	[0.78–1.65]	0.86	0.109	0.88	[0.63–1.24]	0.22
2	C	G	C	C	0.011	0.011	0.010	1.09	[0.36–3.26]	0.94	0.011	1.08	[0.43–2.71]	0.94
3*	C	G	T	C	0.127	0.116	0.115	1.10	[0.74–1.64]	0.97	0.154	1.39	[1.02–1.90]	**0.03**
4*	C	C	T	C	0.410	0.425	0.385	1	ref	ref	0.400	1	ref	ref
5	T	G	C	T	0.327	0.317	0.356	1.26	[0.95–1.67]	0.16	0.325	1.08	[0.85–1.37]	0.73

*P* values for the unadjusted GLM analysis to evaluate the effect of *IL1B* haplotypes on AP and CRC risk among 997 Colombian samples.

*The difference in CRC risk was significant (subnull *p* = 0.04) between *IL1B-1464C* allele carriers and *IL1B-1464G* allele carriers in a conditional haplotype test (controlling for the *IL1B-511*/*IL1B-31* SNPs) and when testing for *IL1B-1464* independent effects (likelihood ratio test: chi-square = 4.38, *df* = 1, *p* = 0.04).

*df*, degrees of freedom.

**Table 2:** Association of *IL1B* haplotypes with AP and CRC risk adjusting for global ancestry and other covariates in Colombian samples.

*IL1B* gene haplotypes			Total (n = 791) Frequency	Controls (n = 343) Frequency	Adenomatous Polyps (AP) (n = 163)							Colorectal Cancer (CRC) (n = 285)						
					Frequency	Unadjusted Model			Adjusted Model			Frequency	Unadjusted Model			Adjusted Model		
N°	(ID)^10^					OR	[95% CI]	p value	OR	[95% CI]	p value		OR	[95% CI]	p value	OR	[95% CI]	p value
1	(a)	CGCT	0.125	0.140	0.126	1.02	[0.67–1.54]	0.94	0.90	[0.57–1.41]	0.65	0.107	0.79	[0.55–1.14]	0.21	0.76	[0.52–1.11]	0.16
2	—	CGCC	0.013	0.016	0.009	0.72	[0.21–2.49]	0.61	0.91	[0.25–3.33]	0.88	0.012	0.79	[0.31–2.01]	0.62	0.61	[0.23–1.61]	0.32
3	(c)	CGTC	0.124	0.105	0.107	1.19	[0.74–1.92]	0.47	1.35	[0.79–2.30]	0.28	0.156	1.55	[1.09–2.20]	**0**.**02**	1.46	**[0**.**99**–**2**.**14]**	0.06
4	(d)	CCTC	0.409	0.426	0.374	1	ref	ref	1	ref	ref	0.409	1	ref	ref	1	ref	ref
5	(b)	TGCT	0.328	0.312	0.383	1.45	[1.05–2.00]	**0.02**	1.40	**[0**.**98**–**2**.**00]**	0.06	0.316	1.05	[0.80–1.36]	0.74	1.02	[0.77–1.34]	0.90

*P* values for the unadjusted and adjusted GLM analyses to evaluate the effect of *IL1B* haplotypes on AP and CRC risk among 791 Colombian samples with available global ancestry estimates and *IL1B* haplotype information.

Within the adjusted models, the OR shows the effect of each haplotype on AP and CRC risk compared to controls after controlling for many covariates, such as sex, age, educational level, global ancestry estimations and array (candidate-gene or genome-wide). According to the anova.haplo.glm() test, including NSAID consumption did not add more information to the model (*p* > 0.05).

**Table 3:** Association between *IL1B* haplotypes and AP or CRC risk among Colombians stratified by region of origin.

*IL1B* gene haplotypes by region	N°	Total (n = 791) Frequency	Controls	Adenomatous Polyps (AP)				Colorectal Cancer (CRC)			
			Frequency	Frequency	OR	[95% CI]	p value	Frequency	OR	[95% CI]	p value
**Andean**											
		(**n = 367**)	(**n = 163**)	(**n = 79**)				(**n = 125**)			
CGCT	1	0.125	0.138	0.127	0.94	[0.51–1.73]	0.83	0.108	0.70	[0.40–1.22]	0.21
CGTC	3	0.067	0.067	0.070	1.31	[0.56–3.06]	0.53	0.064	0.91	[0.45–1.86]	0.80
CCTC	4	0.459	0.463	0.405	1	ref	ref	0.488	1	ref	ref
TGCT	5	0.346	0.328	0.399	1.52	**[0**.**96**–**2**.**40]**	0.08	0.336	0.98	[0.68–1.42]	0.92
**Coastal**											
		(**n = 424**)	(**n = 180**)	(**n = 84**)				(**n = 160**)			
CGCT	1	0.125	0.142	0.125	0.98	[0.54–1.79]	0.95	0.106	0.93	[0.56–1.55]	0.79
CGCC	2	0.022	0.028	0.018	0.93	[0.24–3.62]	0.91	0.019	0.81	[0.29–2.28]	0.69
CGTC	3	0.173	0.139	0.143	1.23	[0.65–2.32]	0.52	0.228	2.06	[1.31–3.25]	**<0**.**01**
CCTC	4	0.366	0.392	0.345	1	ref	ref	0.347	1	ref	ref
TGCT	5	0.312	0.297	0.369	1.52	**[0**.**93**–**2**.**49]**	0.10	0.300	1.14	[0.77–1.69]	0.52

*P* values for the adjusted GLM analyses to evaluate the effect of *IL1B* haplotypes on AP and CRC risk stratified by region of origin.

OR shows the effect of *IL1B* haplotypes on AP and CRC risk adjusted for sex, age and educational level.

**Table 4:** Association of ancestry proportions or *IL1B* risk haplotypes with CRC or AP risk.

CRC risk modelled by African ancestry and the *IL1B-CGTC* haplotype				AP risk modelled by European ancestry and the *IL1B-TGCT* haplotype			
	OR	[95% CI]	p value		OR	[95% CI]	p value
Model 1a				Model 1b			
Global AFR ancestry	1.19	[1.01–1.41]	**0**.**04**	Global EUR ancestry	2.03	[1.27–3.25]	**<0.01**
Model 2a				Model 2b			
Local AFR at *2q14*	4.06	[1.49–11.03]	**0**.**01**	Local EUR at *2q14*	1.91	**[0**.**91**–**4**.**03]**	0.09
Model 3a				Model 3b			
Local AFR at *2q14*	3.40	[1.05–10.98]	**0**.**04**	Local EUR at *2q14*	1.33	[0.60–2.94]	0.48
Global AFR ancestry	1.06	[0.87–1.30]	0.58	Global EUR ancestry	1.94	[1.18–3.17]	**0.01**
Model 4a				Model 4b			
*IL1B-CGTC*	1.58	**[0**.**88**–**2**.**82]**	0.12	*IL1B-TGCT*	1.71	**[0**.**99**–**2**.**96]**	0.06
Model 5a				Model 5b			
*IL1B-CGTC*	1.91	**[0**.**69**–**5**.**27]**	0.21	*IL1B-TGCT*	1.40	**[0**.**79**–**2**.**48]**	0.21
Global AFR ancestry	1.10	**[0**.**89**–**1**.**35]**	0.40	Global EUR ancestry	1.57	**[0**.**78**–**3**.**18]**	0.21
*IL1B-CGTC*: Global AFR ancestry	1.23	[0.81–1.85]	0.33	*IL1B-TGCT*: Global EUR ancestry	1.43	[0.55–3.74]	0.47
Model 6a				Model 6b			
*IL1B-CGTC*	0.50	[0.19–1.28]	0.15	*IL1B-TGCT*	2.18	**[0**.**71**–**6**.**66]**	0.17
Local AFR at *2q14*	1.08	[0.19–6.07]	0.93	Local EUR at *2q14*	2.12	**[0**.**67**–**6**.**64]**	0.20
*IL1B-CGTC*: Local AFR at *2q14*	15.68	[1.23–200.12]	**0**.**03**	*IL1B-TGCT*: Local EUR at *2q14*	0.55	[0.11–2.69]	0.46
Model 7a				Model 7b			
*IL1B-CGTC*	0.94	[0.46–1.92]	0.87	*IL1B-TGCT*	1.48	**[0**.**83**–**2**.**66]**	0.19
Local AFR at *2q14*	3.58	**[0**.**97**–**13**.**30]**	0.06	Local EUR at *2q14*	1.13	[0.49–2.61]	0.77
Global AFR ancestry	1.06	[0.87–1.30]	0.57	Global EUR ancestry	1.91	[1.17–3.13]	**0.01**

*P* values for the adjusted multinomial logistic regression analyses to evaluate the effect of African or European ancestry (global and/or locus-specific) on CRC and AP risk. These models also included the main effects and interactions with copies (0 versus 1 or 2) of the *IL1B* haplotypes of risk (*IL1B-CGTC* for CRC *and IL1B-TGCT* for AP risk). All models are adjusted for sex, age, educational level, NSAID consumption and a family history of CRC.

AFR, African; EUR, European.
