# Supplementary material for: IL1B-CGTC haplotype is associated with colorectal cancer in admixed individuals with increased African ancestry
Source: Sci Rep. 2017 Feb 3;7:41920. doi: 10.1038/srep41920 (PMC5291207; doi:10.1038/srep41920)
Supplement: Supplementary Information [file srep41920-s1.pdf]

IL1B-CGTC haplotype is associated with colorectal cancer in admixed individuals with increased African ancestry

María Carolina Sanabria-Salas[1, 2,*], Gustavo Hernández-Suárez[1], Adriana Umaña-Pérez[2], Konrad Rawlik[3], Albert Tenesa[3,4], Martha Lucía Serrano-López[1, 2], Myriam Sánchez de Gómez[2], Martha Patricia Rojas[1], Luis Eduardo Bravo[5], Rosario Albis[6], José Luis Plata[7], Heather Green[8], Theodor Borgovan[8], Li Li[9],Sumana Majumdar[9], Jone Garai[9], Edward Lee[10], Hassan Ashktorab[10], Hassan Brim[10], Li Li[8], David Margolin[8], Laura Fejerman[11], Jovanny Zabaleta[9,12*].

[1]Subdirección de Investigaciones, Instituto Nacional de Cancerología de Colombia, Bogotá D.C., Colombia; [2]Departamento de Química, Universidad Nacional de Colombia, Bogotá D.C., Colombia; [3]The Roslin Institute and Royal (Dick) School of Veterinary Studies, University of Edinburgh, UK; [4]MRC-Human Genetics Unit, University of Edinburgh, UK; [5]Escuela de Salud Pública, Universidad del Valle, Cali, Colombia; [6]Servicio de Gastroenterología, Instituto Nacional de Cancerología de Colombia, Bogotá D.C., Colombia; [7]Fundación Oftalmológica de Santander, Bucaramanga, Colombia; [8]Ochsner Clinic Foundation, New Orleans, LA, US; [9]Stanley S. Scott Cancer Center, Louisiana State University Health Sciences Center, New Orleans, LA, US; [10]Department of Pathology & Cancer Center, Howard University College of Medicine, Washington D.C., US; [11]Department of Medicine, Division of General Internal Medicine, Institute for Human Genetics and Helen Diller Family Comprehensive Cancer Center, University of California, San Francisco, CA, US;

[12]Department of Pediatrics, Louisiana State University Health Sciences Center, New Orleans, LA, US.

* Corresponding to: jzabal@lsuhsc.edu (J.Z.) or csanabria@cancer.gov.co (M.C.S.S).

Supplementary Information

Supplementary Figures

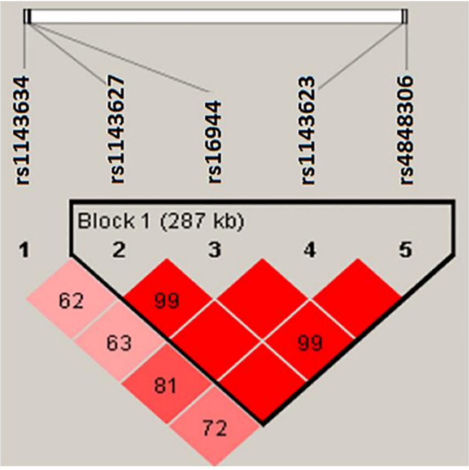

SNP_A	SNP_B	r^2
rs4848306	rs1143623	0.32
rs4848306	rs16944	0.54
rs4848306	rs1143627	0.58
rs1143623	rs16944	0.59
rs1143623	rs1143627	0.56
**rs16944**	**rs1143627**	**0.95**

Supplementary Figure S1 Haplotype block organization of the *IL1B* promoter region.

Each box represents the percentage of LD [D'] between pairs of markers, as generated by Haploview 4.0. D' is color coded, red box [D' 1.00] indicating complete LD. The respective r^2 values between pair of SNPs (A / B) are displayed on the table on the right.

LD, linkage disequilibrium

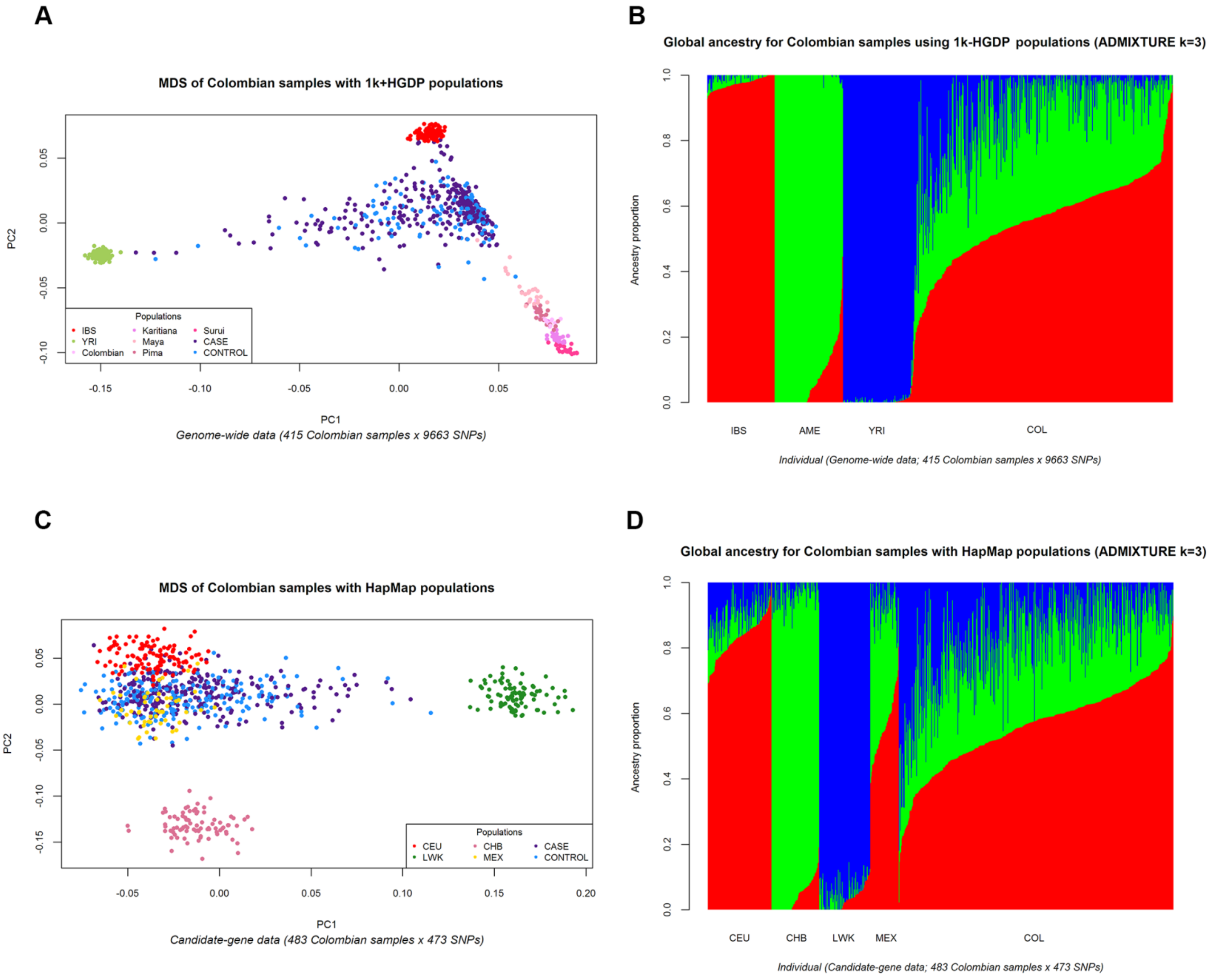
A
MDS of Colombian samples with 1k+HGDP populations
PC2
PC1
0.05
0.00
-0.05
-0.10
-0.15
-0.10
-0.05
0.00
0.05
Populations
IBS
YRI
Colombian
Karitiana
Maya
Pima
Surui
CASE
CONTROL
Genome-wide data (415 Colombian samples x 9663 SNPs)
B
Global ancestry for Colombian samples using 1k-HGDP populations (ADMIXTURE k=3)
Ancestry proportion
0.0
0.2
0.4
0.6
0.8
1.0
IBS
AME
YRI
COL
Individual (Genome-wide data; 415 Colombian samples x 9663 SNPs)
C
MDS of Colombian samples with HapMap populations
PC2
PC1
0.05
0.00
-0.05
-0.10
-0.15
-0.05
0.00
0.05
0.10
0.15
0.20
Populations
CEU
LWK
CHB
MEX
CASE
CONTROL
Candidate-gene data (483 Colombian samples x 473 SNPs)
D
Global ancestry for Colombian samples with HapMap populations (ADMIXTURE k=3)
Ancestry proportion
0.0
0.2
0.4
0.6
0.8
1.0
CEU
CHB
LWK
MEX
COL
Individual (Candidate-gene data; 483 Colombian samples x 473 SNPs)

Supplementary Figure S2 Multidimensional Scaling analyses (MDS) and global ancestry estimates per individual for genome-wide and candidate-gene genotyped samples. A) MDS of the study samples with genome-wide data using reference populations from 1k genomes (IBS for European and YRI for African references) plus HGDP databases (Pima, Maya, Karitiana, Surui and Colombian Native Americans for Amerindian references). B) Corresponding global ancestry estimations per individual for genome-wide genotyped samples. C) MDS of the study samples with candidate-gene data using reference populations from HapMap database (CEU for European, CHB to infer Amerindian and LWK for African references; MEX consist in a Mexican admixed population). D) Corresponding global ancestry estimations per individual for candidate-gene genotyped samples.

1k-HGDP, 1000 genomes plus Human Genome Diversity Project databases; 1k genomes, 1000 genomes database; HGDP, Human Genome Diversity Project database; SNPs, Single Nucleotide Polymorphisms; IBS, Iberian Population in Spain; YRI, Yoruba in Ibadan, Nigeria; AME, includes Pima, Maya, Karitiana, Surui and Colombian reference populations considered as Amerindians; COL, admixed Colombian samples under study; CEU, Utah residents with Northern and Western European ancestry from the CEPH collection; CHB, Han Chinese in Beijing, China; LWK, Luhya in Webuye, Kenya; MEX, Mexican ancestry in Los Angeles, California; CASE, includes adenomatous polyps and colorectal cancer patients from Colombian admixed populations under study; CONTROL, refers to control individuals from Colombian admixed populations under study.

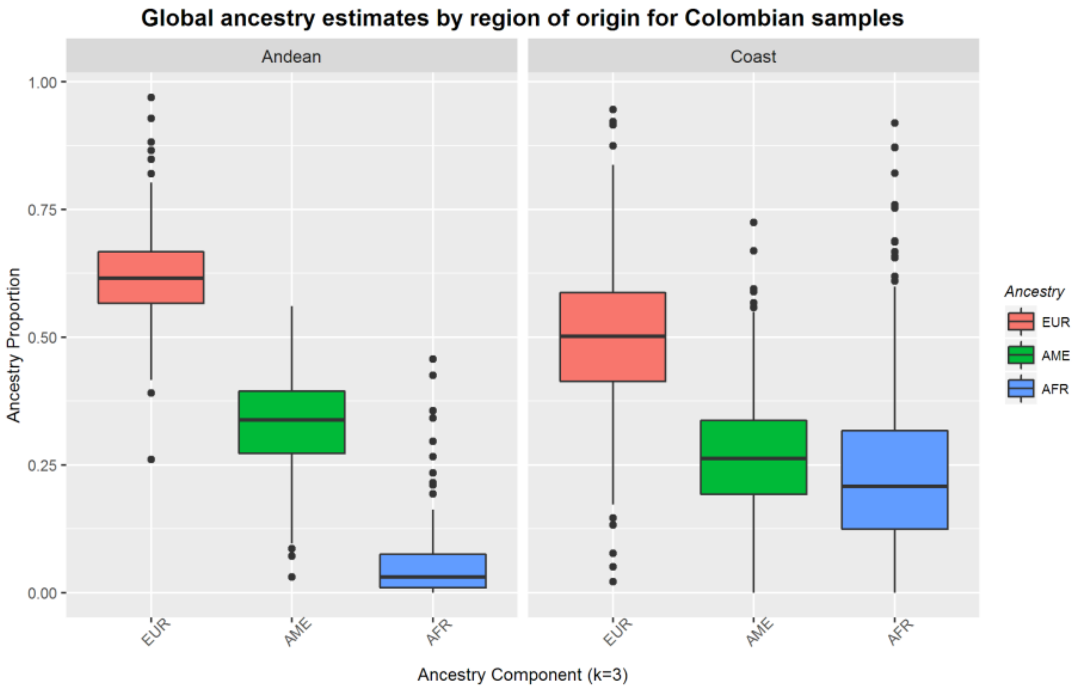

Supplementary Figure S3 Global ancestry estimates by region of origin (Andean or Coastal) for 791 Colombian samples with both ancestry estimations (using candidate-gene or genome-wide data) and *IL1B* haplotype information.

EUR - AME - AFR, refers to the average European, Amerindian and African ancestries from candidate-gene and genome-wide data samples.

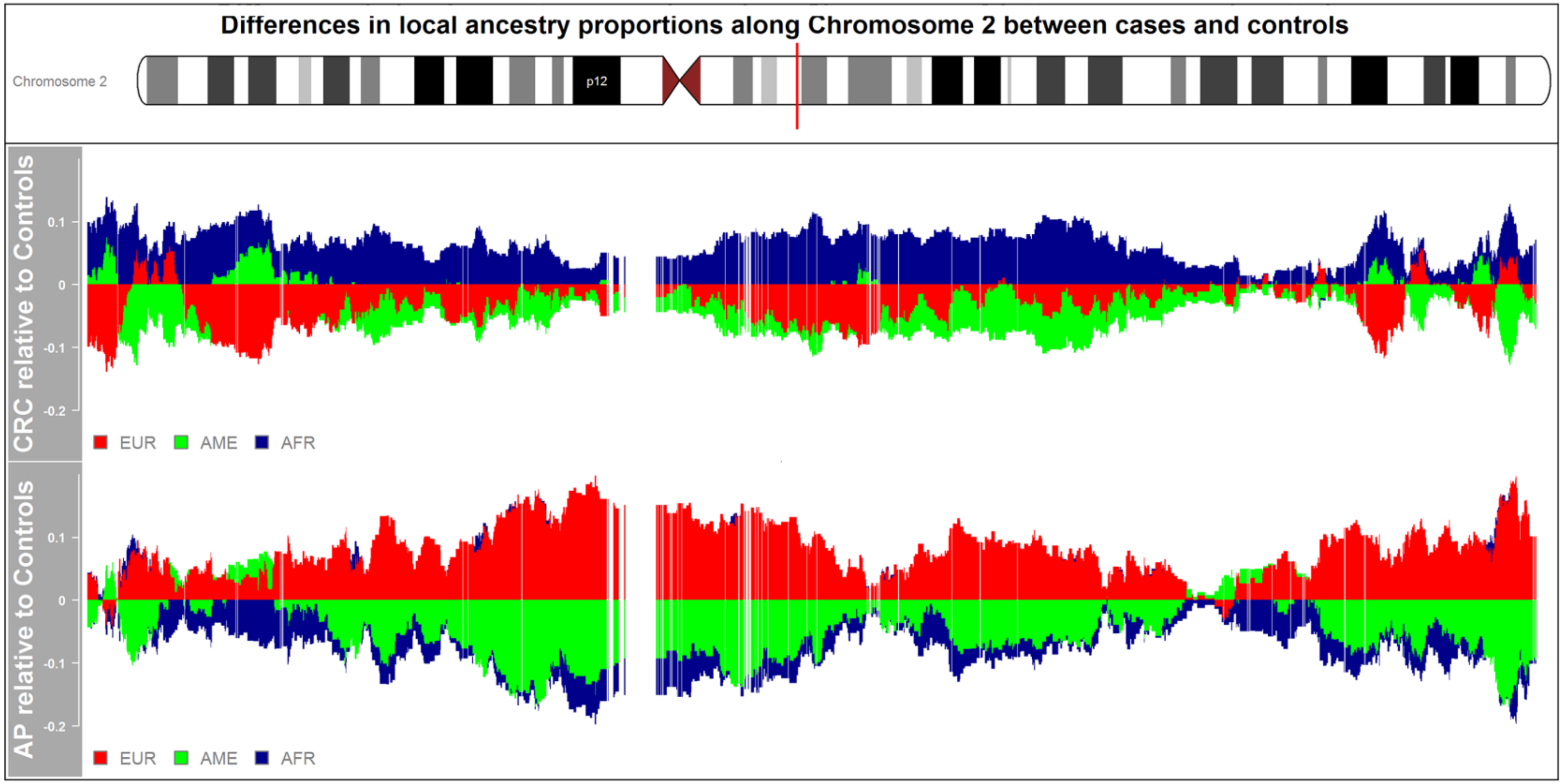

Supplementary Figure S4 Local ancestry estimations along chromosome 2. The red vertical line in chromosome 2 indicates the selected 100000-bp region (Chr2:113500000:113600000; build 37) within locus *2q14* that holds the *IL1B* gene (top panel). A total of 18 SNPs are flanking this region and were used for further locus specific ancestry estimation analyses. The variation of local ancestry proportion at each marker in Chromosome 2 (28579 SNPs) in the CRC and AP groups relative to those in the control group, are displayed in the bottom panel.

CRC, colorectal cancer; AP, adenomatous polyps; EUR - AME - AFR, refers to European, Amerindian and African ancestries

Supplementary Tables

Supplementary Table S1 Characteristics of a Colombian sample of 997 individuals with *IL1B* haplotype information

Characteristics	Controls		Cases					
			Adenomatous Polyps (AP)			Colorectal Cancer (CRC)		
	n=500	(%)	n=191	(%)	p value	n=306	(%)	p value
**Age Range**								
30-39	103	(20.6)	9	(4.7)		18	(5.9)	
40-49	124	(24.8)	30	(15.7)		55	(18.0)	
50-59	125	(25.0)	64	(33.5)		92	(30.1)	
60-69	104	(20.8)	67	(35.1)		103	(33.7)	
70-74	44	(8.8)	21	(11.0)	**<0.01**	38	(12.4)	**<0.01**
**Sex**								
Female	288	(57.6)	97	(50.8)		151	(49.3)	
Male	212	(42.4)	94	(49.2)	0.13	155	(50.7)	**0.03**
**Educational Level**								
No education	8	(1.6)	3	(1.6)		19	(6.2)	
Elementary school	165	(33.1)	50	(26.2)		137	(44.8)	
High School	178	(35.7)	71	(37.2)		98	(32.0)	
Technician	75	(15.0)	21	(11.0)		20	(6.5)	
College degree or higher	73	(14.6)	46	(24.1)	**0.03**	32	(10.5)	**<0.01**
**Family History of CRC**								
No	324	(64.8)	113	(59.2)		205	(67.0)	
Yes	176	(35.2)	78	(40.8)	0.20	101	(33.0)	0.58
**NSAIDs Consumption**								
No	389	(77.8)	151	(79.1)		246	(80.4)	
Yes	111	(22.2)	40	(20.9)	0.80	60	(19.6)	0.43
**Region of Origin**								
Andean	228	(45.6)	88	(46.1)		135	(44.1)	
Coastal	272	(54.4)	103	(53.9)	0.98	171	(55.9)	0.74

P values of the Pearson´s Chi-Squared Test to evaluate for differences in age, sex, educational level, a family history of CRC, NSAID consumption and region of origin by phenotype.

Supplementary Table S2 Genotype and allele frequencies of *IL1B* SNPs among cases and controls

IL1B SNPs	Controls (n=500)	Adenomatous Polyps (AP) (n=191)		Colorectal Cancer (CRC) (n=306)	
	n (%)	n (%)	p value	n (%)	p value
**IL1B-3737C>T**					
**Genotypes**					
C/C	231 (46.2)	74 (38.7)		140 (45.8)	
C/T	220 (44.0)	98 (51.3)		133 (43.5)	
T/T	49 (9.8)	19 (9.9)	0.19	33 (10.8)	0.90
**Alleles**					
C	682 (68.2)	246 (64.4)		413 (67.5)	
T	318 (31.8)	136 (35.6)	0.18	199 (32.5)	0.77
**T carrier**					
Dominant	269/231	117/74	0.08	166/140	0.90
Recessive	49/451	19/172	0.95	33/273	0.65
**IL1B-1464G>C**					
**Genotypes**					
G/G	167 (33.4)	67 (35.1)		113 (36.9)	
G/C	241 (48.2)	101 (52.9)		141 (46.1)	
C/C	92 (18.4)	23 (12.0)	0.13	52 (17.0)	0.59
**Alleles**					
G	575 (57.5)	235 (61.5)		367 (60.0)	
C	425 (42.5)	147 (38.5)	0.18	245 (40.0)	0.33
**C carrier**					
Dominant	333/167	124/67	0.68	193/113	0.31
Recessive	92/408	23/168	**0.04**	52/254	0.61
**IL1B-511C>T**					
**Genotypes**					
C/C	100 (20.0)	45 (23.6)		58 (19.0)	
C/T	258 (51.6)	101 (52.9)		157 (51.3)	
T/T	142 (28.4)	45 (23.6)	0.35	91 (29.7)	0.89
**Alleles**					
C	458 (45.8)	191 (50.0)		273 (44.6)	
T	542 (54.2)	191 (50.0)	0.16	339 (55.4)	0.64
**T carrier**					
Dominant	400/100	146/45	0.30	248/58	0.72
Recessive	142/358	45/146	0.20	91/215	0.68
**IL1B-31T>C**					
**Genotypes**					
T/T	97 (19.4)	44 (23.0)		56 (18.3)	

C/T	254 (50.8)	99 (51.8)		154 (50.3)	
C/C	148 (29.6)	48 (25.1)	0.39	96 (31.4)	0.85
**Alleles**					
T	448 (44.8)	187 (49.0)		266 (43.5)	
C	550 (55.0)	195 (51.0)	0.18	346 (56.5)	0.58
**C carrier**					
Dominant	402/97	147/44	0.29	250/56	0.69
Recessive	148/351	48/143	0.24	96/210	0.61
**IL1B+3954C>T**					
**Genotypes**					
C/C	363 (72.6)	134 (70.2)		225 (73.5)	
C/T	123 (24.6)	52 (27.2)		77 (25.2)	
T/T	14 (2.8)	5 (2.6)	0.78	4 (1.3)	NA
**Alleles**					
C	849 (84.9)	320 (83.8)		527 (86.1)	
T	151 (15.1)	62 (16.2)	0.60	85 (13.9)	0.50
**T carrier**					
Dominant	137/363	57/134	0.52	81/225	NA
Recessive	14/486	5/186	0.90	4/302	NA

P-values are for the genotypic, allelic, dominant and recessive tests. Dominant and recessive models are tests for the variant allele of each *IL1B* SNP.

Supplementary Table S3 Adjusted global ancestry association with AP and CRC risk in Colombian samples

Characteristics	Adenomatous Polyps (AP)			Colorectal Cancer (CRC)		
	**OR**	**[95%CI]**	**p value**	**OR**	**[95%CI]**	**p value**
**Ancestry proportion**						
African ancestry*	1.12	[1.03-1.22]	**0.01**	1.10	[1.03-1.18]	**0.01**
European ancestry*	1.98	[1.35-2.91]	**0.00**	1.00	[0.74-1.36]	0.98
**Sex**						
Female	1	ref	ref	1	ref	ref
Male	1.08	[0.72-1.61]	0.72	1.17	[0.84-1.63]	0.35
**Age**	1.02	[1.00-1.04]	**0.04**	1.01	**[0.99-1.03]**	0.17
**Educational level**						
No education	1	ref	ref	1	ref	ref
Elementary school	0.56	[0.13-2.37]	0.43	0.35	[0.14-0.89]	**0.03**
High School	0.96	[0.23-4.08]	0.96	0.32	[0.12-0.82]	**0.02**
Technician	0.91	[0.20-4.28]	0.91	0.20	[0.07-0.59]	**<0.01**
College degree or higher	1.98	[0.44-8.83]	0.37	0.38	**[0.14-1.08]**	0.07
**NSAIDs consumption**						
No	1	ref	ref	1	ref	ref
Yes	0.76	[0.47-1.23]	0.26	0.65	[0.44-0.97]	**0.04**

P-values for the adjusted multinomial logistic model analysis to evaluate the effect of global ancestry proportions on AP and CRC risk among 791 Colombian samples with available global ancestry estimates and *IL1B* haplotype information, adjusted for sex, age, educational level, NSAID consumption and array (candidate-gene or genome-wide) (best model; Supplementary Table S3.1).

*African and European component logit() transformed.

Supplementary Table 3.1

**Multinomial Logistic Models**	**Df**	**AIC**
Model 1: Pheno ~ Sex	4	1674.966
Model 2: Pheno ~ Age	4	1671.611
Model 3: Pheno ~ Sex + Age	6	1672.566
Model 4: Pheno ~ Sex + Age + Edu	14	1648.631
Model 5: Pheno ~ logit(EUR) + logit(AFR) + Array	8	1598.047
Model 6: Pheno ~ logit(EUR) + logit(AFR) + Array + Sex	10	1599.951
Model 7: Pheno ~ logit(EUR) + logit(AFR) + Array + Sex + Age	12	1600.033
**Model 8: Pheno ~ logit(EUR) + logit(AFR) + Array + Sex + Age + Edu***	**20**	**1583.463**
Model 9: Pheno ~ logit(EUR) + logit(AFR) + Array + Sex + Age + Edu + City	30	1586.007
Model 10: Pheno ~ logit(EUR) + logit(AFR) + Array + Sex + Age + Edu + City + NSAIDs	32	1586.011
Model 11: Pheno ~ logit(EUR) + logit(AFR) + Array + Sex + Age + Edu + City + NSAIDs + Fam_hist	34	1588.753
Model 12: Pheno ~ logit(EUR) + logit(AFR) + Array+ Sex + Age + Edu + Fam_hist	22	1585.383
**Model 13: Pheno ~ logit(EUR) + logit(AFR) + Array + Sex + Age + Edu + NSAIDs***	**22**	**1582.940**

* The best model according to AIC parameter was model 13, followed by model 8.

Pheno, phenotypes; Edu, educational level; logit(EUR), European ancestry logit() transformed; logit(AFR), African ancestry logit() transformed; Array, refers to candidate-gene or genome-wide analyses run in ADMIXTURE software; City, city of origin; NSAID, non-steroid anti-inflammatory drugs; Fam_hist, familial history of CRC; Df, degrees of freedom; AIC, Akaike Information Criteria.

Supplementary Table S4 *IL1B* haplotype frequencies in Colombians, U.S. Black and U.S. White populations

IL1B Haplotypes					This study samples				Chen *et al*[10]	
					Colombian cases and controls			AA CRC cases	ARIC Cohort	
N°	-3737	-1464	-511	-31	Controls (n=500)	AP (n=191)	CRC (n=306)	CRC (n=177)	AA "U.S. Black" (n=227)	"U.S. Non-Hispanic White" (n=900)
					Frequency (%)	Frequency (%)	Frequency (%)	Frequency (%)	Frequency (%)	Frequency (%)
1	C	G	C	T	13.0	13.4	10.9	7.7	14.7	20.0
2	C	G	C	C	1.1	1.0	1.1	4.5	-	-
3*	C	G	T	C	11.6	11.5	15.4	40.8	46.1	6.0
4	C	C	T	C	42.5	38.5	40.0	13.8	10.6	27.7
5**	T	G	C	T	31.7	35.6	32.5	28.6	27.3	44.8

*The CRC risk haplotype (N°3) in our study is the most frequent in AA populations and have the highest *IL1B* gene transcriptional activity, according to Chen et al. 2006[10].

**The AP risk haplotype (N°5) in our study is the most frequent in Caucasians and second in AA populations. According to Chen et al. 2006[10], it exhibits a mid-transcriptional activity of the *IL1B* gene.

AP, adenomatous polyps; CRC, colorectal cancer; AA, African Americans; ARIC, Atherosclerosis Risk Communities Cohort; U.S., United States of America

Supplementary Table S5 *IL1B-511/IL1B-31* haplotype frequencies in Colombian controls, US-Black, US-White and HapMap reference populations

IL1B* simple haplotypes		**Colombian samples**	**HapMap reference populations**				**Chen *et al*[10] ARIC Cohort**	
**-511**	**-31**	**Controls**	**LWK**	**ASW**	**CEU+TSI**	**CHB+JPT**	**AA "U.S. Black"**	**"U.S. Non-Hispanic White"**
T	C	54.1	66.8	53.8	33.9	46.1	56.7	33.7
C	T	44.7	25.0	39.7	61.9	52.6	42.0	64.8
C	C	1.1	7.8	6.3	-	-	-	-

*Simple haplotypes are according to information available in HapMap (only two of the four *IL1B* SNPs tested are included in the listed reference populations). The frequency of simple haplotypes is the sum of related four SNP haplotypes *(IL1B-TC = IL1B-CGTC + IL1B-CCTC; IL1B-CT = IL1B-TGCT + IL1B-CGCT and IL1B-CC = IL1B-CGCC*).

LWK, Luhya in Webuye, Kenya; ASW, African ancestry in Southwest USA; CEU, Utah residents with Northern and Western European ancestry; TSI, Toscani in Italia; CHB, Han Chinese in Beijing, China; JPT, Japanese in Tokyo, Japan; AA, African Americans; ARIC, Atherosclerosis Risk Communities Cohort; U.S., United States of America

Supplementary Methods

Quality control steps

Since two different platforms from Illumina® were used, a candidate-gene array called "*Cancer SNP Panel"* or a genome-wide array called "*Infinium® OmniExpressExome Array",* quality control and pruning steps were performed separately due to large differences in the number of markers tested within each array.

Candidate-gene data: a total of 521 samples were genotyped for 1421 SNPs using this platform. SNPs were excluded from the analysis if there was a significant difference in missing genotype rates among cases and controls (P < 0.01; n = 19), if their minor allele frequency (MAF) was < 0.04 (n = 42), if the SNP overall call failure rate was > 0.05 (n = 48) or if they departed from Hardy-Weinberg equilibrium in controls (P < 0.01; n = 98). Samples were excluded from the analysis if their call failure rates were ≥ 0.03 and/or had heterozygosity rates > 3 SD from the sample mean (n = 26). We also excluded from the analysis one individual of each pair with an identity by descendant (IBD) value > 0.35, thus avoiding duplicates, second-degree relatedness or contaminated samples (n = 12). The panel only includes 13 SNPs on chromosome X; therefore, the *--sex-check* filter was not applied for these samples. After QC a total of 184 unique SNPs were removed, leaving 1237 for further analyses. Also, a total of 38 unique samples were removed after QC steps, leaving 483 samples in the clean database. The pruning step for this QC'd dataset was performed by eliminating one SNP of each pair in linkage disequilibrium (LD) with R^2 > 0.2, in a window size of 50 SNPs and a window shift of 5 SNPs.

Genome-wide data: a total of 443 samples were genotyped for 958178 SNPs using this platform. SNPs were excluded from the analysis if there was a significant difference in missing genotype rates among cases and controls (P < 0.00001; n = 47), if their MAF was < 0.01 (n = 224698), if the SNP overall call failure rate was > 0.05 (n = 14107) or if they departed from Hardy-Weinberg equilibrium in controls (P< 0.00001; n = 187). Samples were excluded from

the analysis if their call failure rates were ≥ 0.03 and/or they had heterozygosity rates > 3 SD from the sample mean (n = 8). Again, one individual of each pair with an IBD value > 0.185 was excluded to avoid duplicates, first and second-degree relatedness or contaminated samples (n = 1). Samples failing the *--sex-check* filter were excluded as recommended (n = 22). After QC a total of 237363 unique SNPs were removed, leaving 720815 for further analyses. Also, a total of 28 unique samples were removed after QC steps, leaving 415 samples in the clean database.

The pruning step for this QC'd dataset was performed as described before but with R^2 > 0.1, in a window size of 50 SNPs and a window shift of 10 SNPs.

Reference populations for genetic structure analysis and global ancestry estimations

For candidate-gene data: due to a lack of enough overlapping markers between this small panel with HGDP Amerindian populations, we chose instead to include reference populations from the HapMap3 project public database (http://hapmap.ncbi.nlm.nih.gov/). We used 473 overlapping SNPs for MDS and ADMIXTURE analyses (Supplementary Figure 1).

For genome-wide data: we included reference population's genotypes from the public databases of 1000 genomes (ftp://ftp.1000genomes.ebi.ac.uk/vol1/ftp/release/20130502) and HGDP (http://www.hagsc.org/hgdp/files.html). Since no filtering of SNPs was previously done according to HGDP site, we filtered it by genotyping rate > 0.05 and MAF < 0.01 in order to avoid low quality SNPs. We used 9663 overlapping SNPs for MDS and ADMIXTURE analyses (Supplementary Figure 1).

Local ancestry inference (LAI) steps

1) The first step consisted in phasing our genome-wide QC´d Colombian sample (~ 700K SNPs) using as reference the already phased genotype information from reference populations in the 1000 genomes database (https://mathgen.stats.ox.ac.uk/impute/1000GP_Phase3.html).

2) Then, we phased the QC´d HGDP files (http://www.hagsc.org/hgdp/files.html) also using as reference the phased 1000 genomes database, and then prepared a reference population's panel by merging the phased data from both databases, now called "phased 1k-HGDP reference panel".

3) Finally, we created input files for RFMix by merging phased Colombian samples from step 1, with selected populations from the previously prepared "phased 1k-HGDP reference panel".
